# A novel risk score to predict deep vein thrombosis after spontaneous intracerebral hemorrhage

**DOI:** 10.3389/fneur.2022.930500

**Published:** 2022-10-28

**Authors:** Ruijun Ji, Linlin Wang, Xinyu Liu, Yanfang Liu, Dandan Wang, Wenjuan Wang, Runhua Zhang, Ruixuan Jiang, Jiaokun Jia, Hao Feng, Zeyu Ding, Yi Ju, Jingjing Lu, Gaifen Liu, Yongjun Wang, Xingquan Zhao, Beijing Registration of Intracerebral Hemorrhage investigators

**Affiliations:** ^1^Department of Neurology, Tiantan Hospital, Capital Medical University, Beijing, China; ^2^China National Clinical Research Center for Neurological Diseases, Beijing, China; ^3^Center of Stroke, Beijing Institute for Brain Disorders, Beijing, China; ^4^Beijing Key Laboratory of Translational Medicine for Cerebrovascular Disease, Beijing, China; ^5^Beijing Key Laboratory of Brain Function Reconstruction, Beijing, China

**Keywords:** intracerebral hemorrhage, deep vein thrombosis, risk model, discrimination, calibration

## Abstract

**Background and purpose:**

Studies showed that patients with hemorrhagic stroke are at a higher risk of developing deep vein thrombosis (DVT) than those with ischemic stroke. We aimed to develop a risk score (intracerebral hemorrhage-associated deep vein thrombosis score, ICH-DVT) for predicting in-hospital DVT after ICH.

**Methods:**

The ICH-DVT was developed based on the Beijing Registration of Intracerebral Hemorrhage, in which eligible patients were randomly divided into derivation (60%) and internal validation cohorts (40%). External validation was performed using the iMCAS study (In-hospital Medical Complication after Acute Stroke). Independent predictors of in-hospital DVT after ICH were obtained using multivariable logistic regression, and β-coefficients were used to generate a scoring system of the ICH-DVT. The area under the receiver operating characteristic curve (AUROC) and the Hosmer–Lemeshow goodness-of-fit test were used to assess model discrimination and calibration, respectively.

**Results:**

The overall in-hospital DVT after ICH was 6.3%, 6.0%, and 5.7% in the derivation (*n* = 1,309), internal validation (*n* = 655), and external validation (*n* = 314) cohorts, respectively. A 31-point ICH-DVT was developed from the set of independent predictors including age, hematoma volume, subarachnoid extension, pneumonia, gastrointestinal bleeding, and length of hospitalization. The ICH-DVT showed good discrimination (AUROC) in the derivation (0.81; 95%CI = 0.79–0.83), internal validation (0.83, 95%CI = 0.80–0.86), and external validation (0.88; 95%CI = 0.84–0.92) cohorts. The ICH-DVT was well calibrated (Hosmer–Lemeshow test) in the derivation (*P* = 0.53), internal validation (*P* = 0.38), and external validation (*P* = 0.06) cohorts.

**Conclusion:**

The ICH-DVT is a valid grading scale for predicting in-hospital DVT after ICH. Further studies on the effect of the ICH-DVT on clinical outcomes after ICH are warranted.

## Introduction

Spontaneous intracerebral hemorrhage (ICH) accounts for approximately 15% to 20% of all strokes and is one of the leading causes of mortality and morbidity worldwide (1, 2). Despite advances in medical knowledge, the treatment of ICH remains strictly supportive with not many evidence-based interventions currently available (3, 4).

Venous thromboembolism (VTE) is a common and potentially life-threatening complication after stroke (5). VTE includes deep vein thrombosis (DVT) and pulmonary embolism (PE). The former is the most prevalent presentation, and the latter is the most severe form of VTE (6). Studies have indicated that patients with hemorrhagic stroke are at significantly higher risk of DVT than those with ischemic stroke (7–10). DVT prophylaxis might be a potential target to improve clinical outcomes after ICH. In addition, the optimal approach for DVT prophylaxis in an ICH patient is a challenge of balancing the reduction in the incidence of DVT and pulmonary embolus (PE) without risking an increase in catastrophic hemorrhages.

Several risk factors for DVT after stroke have been identified, such as age (11–15), gender (11–13, 16), race (11, 12, 17), heart failure (8), atrial fibrillation (7, 18), hemiparesis (13–15), immobility (13, 19), disorder of consciousness (8), stroke severity (7, 14), stroke subtypes (7, 13, 15), infections (20–22), hematoma volume (14), and length of hospital stay (7, 22, 23). However, no reliable scoring system is currently available to predict in-hospital DVT after ICH in routine clinical practice or clinical trials. An effective risk stratification model for in-hospital DVT after ICH would be helpful to identify high-risk patients and implement tailored preventive strategies. In addition, for clinical trials, it could be used in nonrandomized studies to control for case-mix variation and in controlled studies as a selection criterion.

In the study, we aimed to derivate and validate a clinical score (intracerebral hemorrhage-associated deep vein thrombosis score, ICH-DVT score) for predicting in-hospital DVT after ICH following the TRIPOD (Transparent Reporting of a multivariable prediction model for Individual Prognosis Or Diagnosis) guideline (24).

## Methods

### Derivation and validation cohorts

The derivation and internal validation cohorts were derived from the Beijing Registration of Intracerebral Hemorrhage, which was a multicenter, prospective, and observational cohort study. Thirteen hospitals in Beijing area participated in the study. To be eligible for the study, subjects had to meet the following criteria: (1) age 18 years or older; (2) hospitalized with a primary diagnosis of spontaneous ICH confirmed by brain CT or MRI; (3) time from stroke onset to hospital admission of < 24 h; and (4) written informed consent from patients or their legal representatives. The study protocol was approved by the Institutional Review Board (IRB) of the Beijing Tiantan Hospital (KY2014-023-02). The eligible patients were randomly divided into derivation cohort (60%) and internal validation cohort (40%).

The external validation cohort was based on the iMCAS study (In-hospital Medical Complication after Acute Stroke) (7), which is a prospective registry of stroke patients admitted to Beijing Tiantan Hospital from January 2014 to December 2016. To be eligible for the iMCAS, subjects had to meet the following criteria: (1) age 18 years or older; (2) hospitalized with a primary diagnosis of AIS, ICH, or SAH confirmed by brain CT or MRI; (3) time from stroke onset to hospital admission of < 7 days; and (4) written informed consent from patients or their legal representatives. The iMCAS was approved by the Ethics Committee of Beijing Tiantan Hospital. For this study, only patients with ICH were included.

### Data collection and definition of variables

Standardized electronic case report forms were used for data collection in both the Beijing Registration of Intracerebral Hemorrhage and iMCAS. For the study, the following candidate variables were included and analyzed: (1) demographics; (2) time from onset to hospital; (3) stroke risk factors; (4) pre-admission antithrombotic medications; (5) pre-stroke modified Rankin scale (mRS) score (this information is obtained from patients or their legal representatives); (6) National Institutes of Health Stroke Scale (NIHSS) score and Glasgow Coma Scale (GCS) score on admission; (7) admission systolic and diastolic blood pressure (mmHg); (8) admission laboratory tests; (9) neuroimaging variables: intracerebral hemorrhage volume (measured using the ABC/2 method), hematoma location (supratentorial or infratentorial ICH), intraventricular extension (presence or absence), and subarachnoid extension (presence or absence); (10) etiology diagnosis (primary or secondary ICH); (11) ambulation within 48 h after admission; (12) DVT prophylaxis within 48 h after admission [intermittent pneumatic compression (ICP) vs. anticoagulation (unfractionated heparin, low-molecular-weight heparin, or non-vitamin K antagonist oral anticoagulants)]; (13) surgical treatment (craniotomy evacuation, minimally invasive surgical therapy, or brain ventricle puncture and drainage); (14) withdrawal of medical care; (15) in-hospital pneumonia after ICH; (16) in-hospital gastrointestinal bleeding (GIB) after ICH; and (17) length of hospital stay (LOS).

### Diagnosis of in-hospital DVT after ICH

In this study, in-hospital DVT was diagnosed by the treating physicians based on clinical manifestations, such as swelling, pitting edema, redness, tenderness, and presence of collateral superficial veins, and D-dimer and verified by sequential compression Doppler ultrasound. Only DVT that developed after hospital admission was counted.

### Statistical analysis

Categorical variables were expressed as proportions. Continuous variables were expressed as mean and standard deviation (SD) or median and interquartile range (IQR). Chi-square or Fisher's exact test was used to compare categorical variables between groups, and Mann–Whitney test or independent *t*-test was employed to compare continuous variables between groups.

Model building was performed exclusively in the derivation cohort. In univariate analysis, Mann–Whitney test was employed to compare continuous variables and Chi-square test was used to compare categorical variables. A multivariable logistic regression with stepwise backward was performed to determine independent predictors of in-hospital DVT after ICH. Candidate variables were those with biologically plausible link to DVT after ICH on the basis of prior publication and those associated with in-hospital DVT after ICH in univariate analysis (*P* < 0.1). The tolerance and variance inflation factor (VIF) were calculated to test collinearity between the predictors of final multivariable model. The β-coefficients of predictors from the final model were used to generate a scoring system of the ICH-DVT. To derive an integer value for each predictor, the β-coefficients were multiplied by 4 and were rounded to the closest integer. The resulting ICH-DVT was validated by assessing model discrimination and calibration. Discrimination was assessed by calculating the area under the receiver operating characteristic curve (AUROC). Meanwhile, sensitivity, specificity, positive predictive value (PPV), and negative predictive value (NPV) were calculated at the maximum Youden index. Calibration was assessed by plotting the observed vs. predicted risk according to 10 deciles of the predicted risk. In addition, the Hosmer–Lemeshow goodness-of-fit test was performed and the Snell R-square and Nagelkerke R-square were calculated.

All tests were two-tailed, and statistical significance was determined at an α level of 0.05. Statistical analysis was performed using SAS 9.1 (SAS Institute, Cary, NC, USA), SPSS 21.0 (SPSS Inc., Chicago, IL, USA), and MedCalc 12.3 software (MedCalc ^®^, Belgium).

## Results

### Baseline characteristics

The baseline characteristics of the derivation and validation cohorts are listed in Table 1. From December 2014 to September 2016, a total of 1,964 patients were enrolled in the Beijing Registration of Intracerebral Hemorrhage. The mean age was 56.8 ± 14.4, and 67.6% were male. The median time from onset to hospital was 4.0 hours (IQR: 1.90–11.1). The median GCS and NIHSS score on admission was 14 (IQR: 8–15) and 11 (IQR: 3–21), respectively. The median LOS was 16 days (IQR: 8–22). A total of 122 (6.2%) patients were diagnosed with in-hospital DVT after ICH. The eligible patients were randomly divided into derivation cohort (60%, *n* = 1,309) and internal validation cohort (40%, *n* = 655), which were well matched with regard to baseline characteristics and an overall rate of in-hospital DVT after ICH (Table 1).

**Table 1:** Baseline characteristics.

	**Overall cohort (*n* = 1,964)**	**Derivation cohort (*n* = 1,309)**	**Internal validation cohort (*n* = 655)**	***P_1_* value**	**External validation cohort (n = 314)**
Demographics					
Age, y, median (IQR)	56.8 ± 14.4	56.8 ± 14.6	56.9 ± 13.9	0.19	54.7 + 14.2
Gender (male), *n* (%)	1,327 (67.6)	866 (67.7)	441 (67.3)	0.87	221 (70.4)
Onset to hospital (hours), median (IQR)	4.0 (1.90–11.0)	4.0 (1.92–11.0)	3.9 (1.97–11.0)	0.76	78 (24–96)
Risk factors, *n* (%)					
Hypertension	1,367 (69.6)	908 (69.4)	459 (70.1)	0.75	208 (66.9)
Diabetes mellitus	289 (14.7)	196 (15.0)	93 (14.2)	0.65	41 (13.1)
Dyslipidemia	184 (9.4)	109 (8.3)	75 (11.5)	0.03	36 (11.5)
Atrial fibrillation	30 (1.5)	20 (1.5)	10 (1.5)	0.99	10 (3.2)
History of stroke/TIA	309 (15.7)	208 (15.9)	101 (15.4)	0.79	48 (15.3)
Myocardial infarction	38 (1.9)	20 (1.5)	18 (2.7)	0.06	26 (8.3)
Heart failure	8 (0.4)	6 (0.5)	2 (0.3)	0.62	3 (1.0)
Current smoker	628 (32.0)	403 (30.8)	225 (34.4)	0.11	120 (38.2)
Alcohol consumption	716 (36.5)	470 (35.9)	246 (37.6)	0.47	166 (52)
Pre-admission anticoagulation, *n* (%)	21 (1.1)	14 (1.1)	7 (1.1)	0.99	5 (1.6)
Pre-admission antiplatelet, *n* (%)	277 (14.1)	181 (13.8)	96 (14.7)	0.62	25 (7.9)
Pre-stroke mRS score, median (IQR)	0 (0–0)	0 (0–0)	0 (0–0)	0.36	0 (0–0)
Admission NIHSS score, median (IQR)	11 (3–21)	11 (3–21)	11 (4–21)	0.89	4 (1–10)
Admission GCS score, median (IQR)	14 (8–15)	14 (8–15)	14 (9–15)	0.26	15 (14–15)
Admission dysphagia, *n* (%)	666 (33.9)	441 (33.7)	225 (34.4)	0.77	24 (7.6)
Admission SBP (mm Hg), median (IQR)	165 (147–186)	164 (146–186)	167 (150–187)	0.10	158 (140–171)
Admission DBP (mm Hg), median (IQR)	96 (82–109)	95 (81–108)	98 (84–110)	0.10	93 (83–104)
Admission WBC, 10^9^/L, median (IQR)	9.79 (7.35–13.0)	9.68 (7.29–12.9)	10.0 (7.56–13.0)	0.26	8.83 (7.34–11.0)
Admission glucose (mmol/L), median (IQR)	7.31 (6.08–9.20)	7.26 (6.05–9.10)	7.49 (6.13–9.40)	0.20	5.04 (4.37–6.07)
Admission creatinine (μmol/L), median (IQR)	63.4 (52.7–77.0)	63.1 (52.3–76.6)	63.9 (53.8–77.0)	0.17	61.7 (52.1–72.1)
Hematoma location				0.91	
Supratentorial ICH, *n* (%)	1,752 (89.2)	1,167 (89.2)	585 (89.3)		282 (89.8)
Infratentorial ICH, *n* (%)	212 (10.8)	142 (10.8)	70 (10.7)		32 (10.2)
Hematoma volume (cm^3^), median (IQR)	15.8 (6.0–38.6)	15.5 (5.9–37.0)	16.7 (6.6–40.0)	0.20	15 (10–30)
Intraventricular extension, *n* (%)	655 (33.4)	430 (32.8)	225 (34.4)	0.51	109 (34.7)
Subarachnoid extension, *n* (%)	264 (13.4)	182 (13.9)	82 (12.5)	0.39	30 (9.6)
Etiology diagnosis, *n* (%)				0.86	
Primary ICH	1,785(90.9)	1,193 (91.1)	592 (90.4)		277 (88.2)
Secondary ICH	159 (8.1)	103 (7.3)	56 (8.5)		34 (10.8)
Primary IVH	20 (1.0)	13 (1.0)	7 (1.1)		…
Ambulatory within 48 h after admission, n (%)	467 (23.8)	318 (24.3)	149 (22.7)	0.47	…
DVT prophylaxis within 48 h after admission					
ICP	96 (4.9)	69 (5.3)	27 (4.1)	0.32	112 (35.7)
Anticoagulation	5 (0.3)	4 (0.2)	1 (0.8)	0.46	…
Withdrawal of medical care, *n* (%)	139 (7.1)	99 (7.6)	40 (6.1)	0.24	21 (6.7)
Surgical treatment, *n* (%)	366 (18.6)	251 (19.2)	115 (17.6)	0.39	43 (13.7)
Length of hospital stay, median (IQR)	16 (8–22)	16 (9–22)	16 (8–22)	0.99	14 (12–18)
In-hospital pneumonia, *n* (%)	575 (29.3)	390 (29.8)	185 (28.2)	0.49	59 (18.8)
In-hospital GIB, *n* (%)	194 (9.9)	128 (9.8)	66 (10.1)	0.87	20 (6.4)
In-hospital DVT, *n* (%)	122 (6.2)	83 (6.3)	39 (6.0)	0.73	18 (5.7)

IQR, interquartile range; TIA, transient ischemic attack; mRS, modified Rankin scale; NIHSS, National Institutes of Health Stroke Scale score; GCS, Glasgow Coma Scale; SBP, systolic blood pressure; DBP, diastolic blood pressure; WBC, white cell count; ICH, intracerebral hemorrhage; IVH, intraventricular hemorrhage; ICP, intermittent pneumatic compression; GIB, gastrointestinal bleeding; DVT, deep vein thrombosis.

A total of 314 patients with ICH in the iMCAS were included for external validation. The mean age was 54.7 ± 14.2, and 70.4% were male. The median time from onset to hospital was 3 days (IQR: 1–4 days). The median NIHSS and GCS scores on admission were 4 (IQR: 1–10) and 15 (IQR: 14–15), respectively. The median LOS was 14 days (IQR: 12–18). A total of 18 (5.7%) patients were diagnosed with in-hospital DVT after ICH (Table 1).

### Predictors of in-hospital DVT after ICH

The results of univariate analysis for predictors of in-hospital DVT after ICH in the derivation cohort are given in Supplementary Table 1, and the multivariable predictors are listed in Table 2. Age (*P* < 0.001), hematoma volume (*P* = 0.01), subarachnoid extension (*P* < 0.001), pneumonia (*P* < 0.001), gastrointestinal bleeding (*P* = 0.003), and length of hospitalization (*P* < 0.001) were significantly associated with in-hospital DVT after ICH. The tolerance of covariates in the final model ranged between 0.81 and 0.98, and the VIF ranged between 1.02 and 1.23.

**Table 2:** Multivariable predictors of in-hospital DVT after ICH in the derivation cohort (*n* = 1,309).

**Variables**	**β-coefficients**	**SE**	adjusted OR*	**95% CI**	***P***
Model intercept	−4.913				
Age (1-year increase)	0.025	0.007	1.03	1.01–1.04	< 0.001
Hematoma volume (1-ml increase)	0.006	0.003	1.01	1.00–1.01	0.01
Subarachnoid extension (yes)	0.874	0.238	2.39	1.3503.82	< 0.001
Occurrence of pneumonia (yes)	1.034	0.223	2.81	1.82–4.36	< 0.001
Occurrence of GIB (yes)	0.748	0.253	2.11	1.29–3.47	0.003
Length of hospitalization (1-day increase)	0.018	0.004	1.02	1.00–1.03	< 0.001

^*^Multivariable logistic regression adjusted for demographics, time from onset to hospital, stroke risk factors, pre-admission antithrombotic medications, pre-stroke dependence, admission NIHSS and GCS scores, blood pressure, blood glucose, hematoma volume, location, intraventricular and subarachnoid extension, etiology, ambulation within 48h after admission, DVT prophylaxis within 48 hours after admission, surgical treatment, withdrawal of medical care, in-hospital medical complications, and length of hospital stay.

DVT, deep vein thrombosis; ICH, intracerebral hemorrhage; SE, standard error; OR, odds ratio; CI, confidence interval; NIHSS, National Institutes of Health Stroke Scale; GCS, Glasgow Coma Scale; GIB, gastrointestinal bleeding.

### Derivation of the ICH-DVT

**Figure 1:**
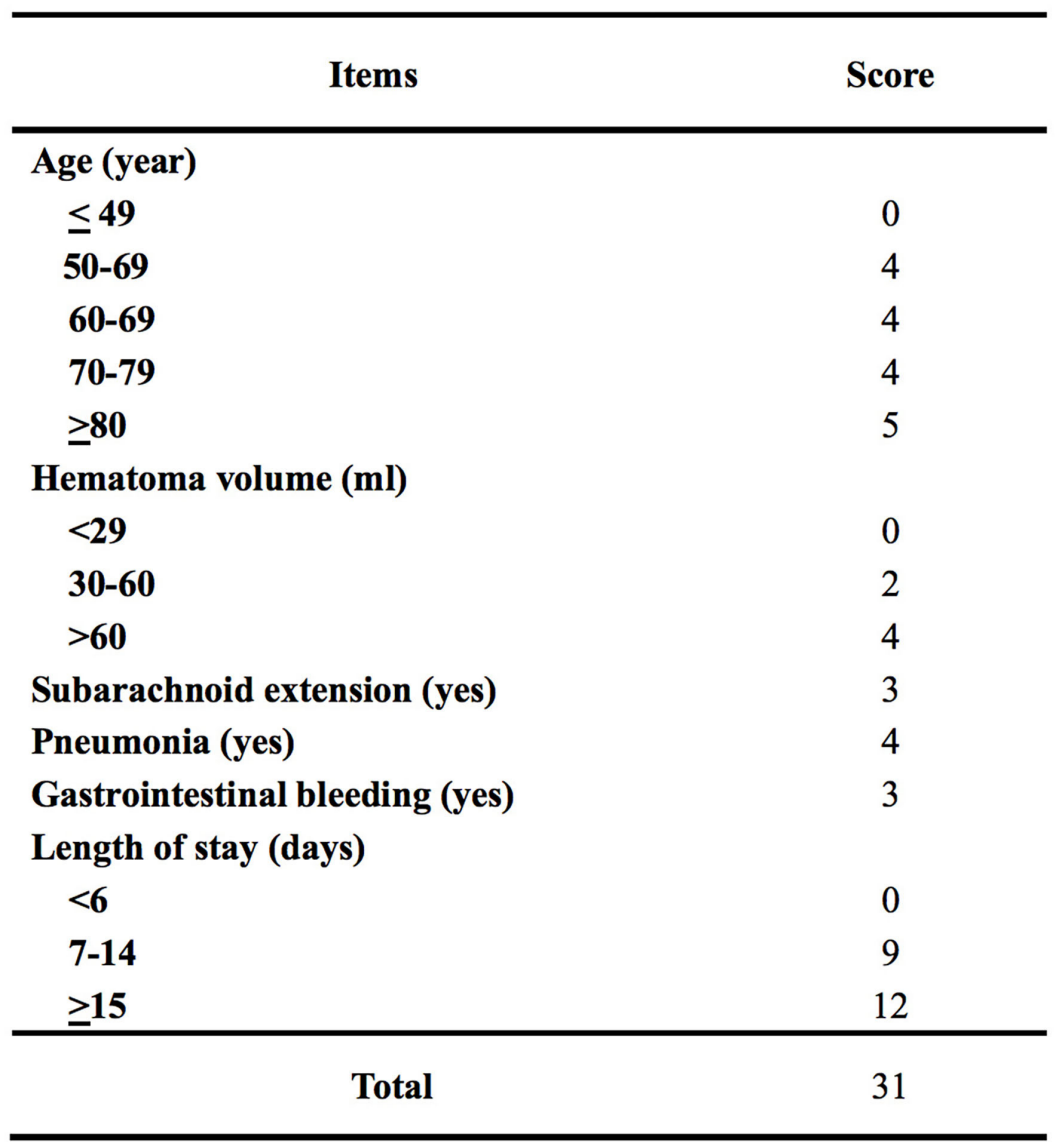
Scoring system of intracerebral hemorrhage-associated deep vein thrombosis score (the ICH-DVT score).

**Figure 2:**
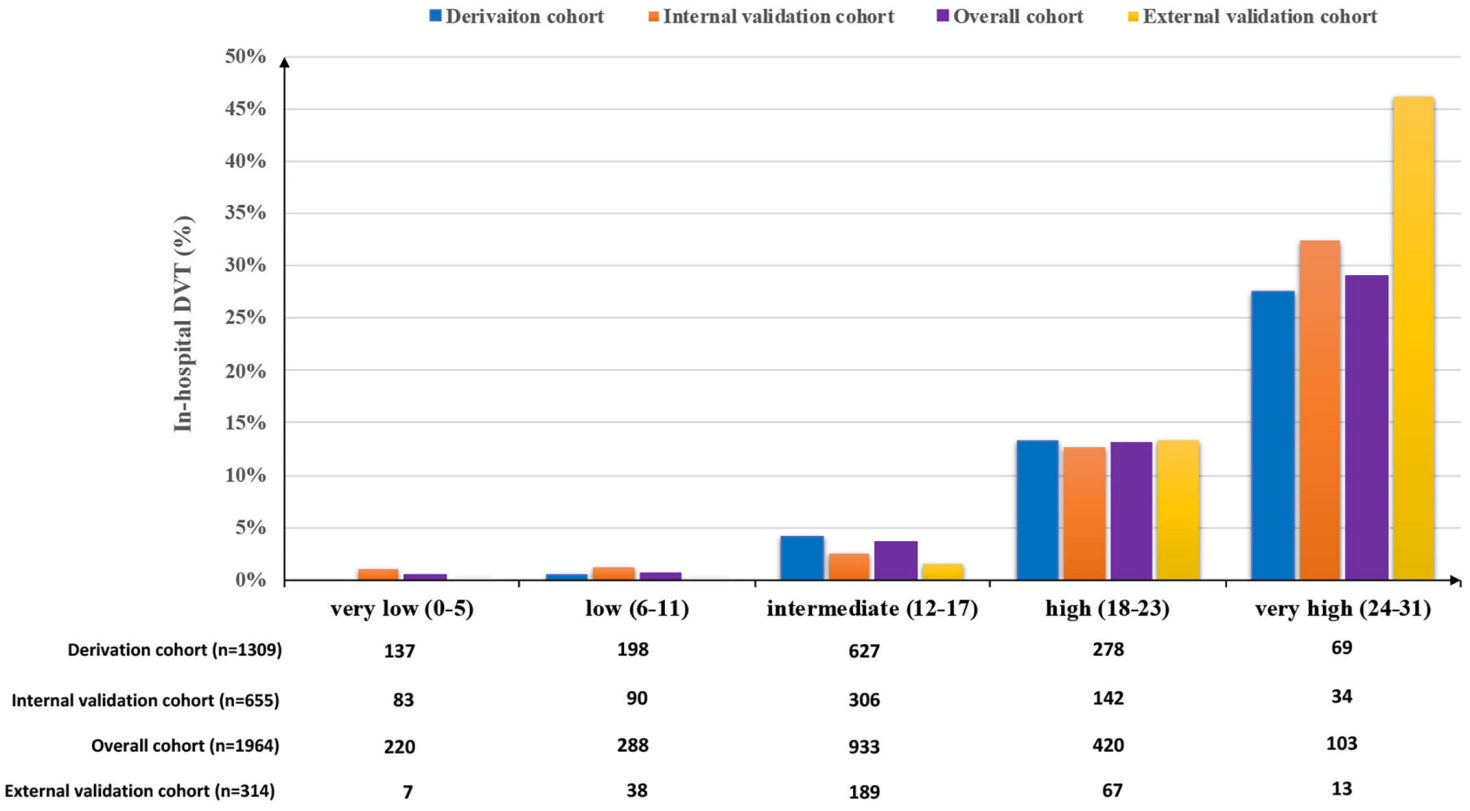
Proportion of in-hospital DVT after ICH according to the ICH-DVT score in the derivation, internal validation, and external validation cohorts. The risk categories were assigned in six-point increments. The potential risk of in-hospital DVT after ICH increased steadily with a higher ICH-DVT score.

The β-coefficients of predictors of the final multivariable model were used to generate a scoring system of the ICH-DVT. To derive an integer value for each predictor, the β-coefficients were multiplied by 4 and were rounded to the closest integer. The scoring system of the ICH-DVT is shown in Figure 1. The risk categories were assigned in six-point increments, and the magnitude of the score had predictive implication. The risk of in-hospital DVT after ICH increased steadily with a higher ICH-DVT score (Figure 2). Due to that, it is hard to clarify whether patients with a longer length of stay are more likely to develop DVT or whether occurrence of DVT leads to a longer hospitalization. We established a risk model without LOS (Supplementary Table 3).

### Internal validation of the ICH-DVT

The predictive performance (AUROC) of the ICH-DVT in the derivation (*n* = 1,309) and internal validation cohorts (*n* = 655) was 0.81 (95%CI = 0.79–0.83) and 0.83 (95%CI = 0.80–0.86), respectively (Table 2). The predicted and observed risks of in-hospital DVT after ICH were in close agreement according to 10 deciles of predicted risk in the derivation and internal validation cohorts (Supplementary Figure 1). The Hosmer–Lemeshow test was not significant in derivation (*P* = 0.53), internal validation (*P* = 0.38), and overall (*P* = 0.61) cohorts. The Snell R-square and Nagelkerke R-square of the Hosmer–Lemeshow goodness-of-fit test in the internal validation cohort were 0.08 and 0.22, respectively (Supplementary Table 2).

### External validation of the ICH-DVT

In the external validation cohort (*n* = 314), the ICH-DVT showed good discrimination with an AUROC of 0.88 (95%CI = 0.84–0.92) (Table 2). The plot of observed vs. predicted risk of in-hospital DVT after ICH showed a high correlation between the observed and predicted risks in the external validation cohort (Supplementary Figure 1). The Hosmer–Lemeshow test was not significant (*P* = 0.06). The Snell R-square and Nagelkerke R-square of the Hosmer–Lemeshow goodness-of-fit test were 0.11 and 0.32, respectively (Supplementary Table 2).

## Discussion

In the study, we aimed to derive and validate a risk score for predicting in-hospital DVT after ICH. Age, hematoma volume, subarachnoid extension, pneumonia, GIB, and length of hospitalization were predictive of in-hospital DVT after ICH. A 31-point ICH-DVT score was developed from the set of independent predictors, which showed good discrimination and calibration in the derivation, internal validation, and external validation cohorts.

Several risk factors have been identified for in-hospital DVT after stroke. Consistent with these studies, we found that in-hospital DVT after ICH was significantly associated with age, hematoma volume, subarachnoid extension, pneumonia, GIB, and length of hospitalization. Previous studies showed that pneumonia was significantly associated with in-hospital DVT after stroke (20, 21). Similar results were verified in both ischemic and hemorrhagic stroke (21). Patients with GIB are at increased risk of developing venous thromboembolism (25). In addition, a study showed an increased risk of thromboembolic events in patients whose anticoagulation was stopped after hospitalization for index GIB (26). Organ crosstalk is an emerging, interesting, and clinically relevant field. Currently, little is known about the pathophysiological mechanisms of medical complications crosstalk after acute stroke. A study indicated that pneumonia might play an important role in the development of several non-pneumonia medical complications (including DVT) after acute stroke (21). There would be a sequential response involving activation of the coagulation cascade, platelet plug formation, and upregulation of endogenous defense mechanisms after hemorrhagic stroke (27–29). Similarly, we speculated that activation of endogenous coagulation system might play an important role in the association between GIB and risk of in-hospital DVT after ICH. Further studies to clarify the molecular mechanisms underlying the interrelationship between pneumonia, GIB, and DVT after ICH are warranted.

When assessing model discrimination, the ICH-DVT showed good predictive performance with regard to in-hospital DVT after ICH in the derivation, internal validation, and external validation cohorts (Table 3). In addition, the ICH-DVT score was well calibrated in the derivation, internal validation, and external validation cohorts (Supplementary Table 2). It was noteworthy that the ICH-DVT score had higher NPV than PPV for in-hospital DVT after ICH (Table 3), which meant that lower values more consistently predict patients without in-hospital DVT than higher values that predict those developing in-hospital DVT after ICH. Development of future models might benefit from attempts to make them more balanced in this regard.

**Table 3:** Discrimination of ICH-DVT with regard to in-hospital DVT after ICH.

	**AUROC**	**95% CI**	***P* value^&^**	**Youden index**	**Cutoff**	**Sensitivity**	**Specificity**	**PPV**	**NPV**
In the derivation cohort (*n* = 1,309)	0.81	0.79–0.83	< 0.0001	0.463	16	0.711	0.733	0.153	0.974
In the internal validation cohort (*n* = 655)	0.83	0.80–0.86	< 0.0001	0.537	16	0.795	0.742	0.163	0.983
In the overall cohort (*n* = 1,964)	0.82	0.80–0.83	< 0.0001	0.474	16	0.738	0.736	0.156	0.977
In the external validation cohort (*n* = 314)	0.88	0.84–0.92	< 0.0001	0.688	16	0.944	0.743	0.183	0.995

DVT, deep vein thrombosis; ICH, intracerebral hemorrhage; AUROC, area under the receiver operating characteristic curve; CI, confidence interval; PPV, positive predictive value; NPV, negative predictive value.

DVT prophylaxis after ICH is highly recommended by clinical guidelines from different countries (3, 4, 30, 31). A study showed that the median time from onset to diagnosis of DVT after ICH was 7 days (IQR = 4–9) (7). Therefore, the first week after onset might be a critical time window for preventing DVT after ICH. According to the AHA/ASA guidelines for ICH management, patients with ICH should have intermittent pneumatic compression for the prevention of VTE beginning the day of hospital admission (Class I; Level of Evidence A). After documentation of cessation of bleeding, low-dose subcutaneous low-molecular-weight heparin or unfractionated heparin may be considered for the prevention of VTE in patients with a lack of mobility after 1–4 days from onset (Class IIb; Level of Evidence B) (3). There can be difficulty in balancing the increased risk of further intracranial hemorrhage vs. the benefit of starting anticoagulation to prevent VTE in daily clinical practice. The ICH-DVT score could be helpful to identify high-risk patients of developing in-hospital DVT after ICH, which would be useful for implementing tailored preventive strategies. In addition, for clinical trials, ICH-DVT could be used in nonrandomized studies to control for case-mix variation and in controlled studies as a selection criterion. Randomized controlled trials on efficacy of DVT prophylaxis and ICH outcomes with stratification of patients' potential risk are warranted. Clinical trials conducted in this way would allow clarifying more accurately which prevention strategies will work in which risk stratification patients.

Clinical practice of DVT prophylaxis after stroke is considerably variable and practitioner dependent (32). We recommended R–P–R (risk–prevention–reassessment) model to prevent in-hospital DVT after ICH. The R–P–R model could be summarized in three steps: Step 1 (risk): to stratify potential risk of developing DVT by using the ICH-DVT; Step 2 (prevention): to apply tailed preventive strategies based on a potential risk of in-hospital DVT and hemorrhagic events. Therapeutic decision (pharmacologic vs. mechanical prophylaxis) could be based on an individual benefit–risk ratio assessment. Pharmacologic agents are the preferred agents for prophylaxis as they reduce VTE more effectively than mechanical prophylaxis (33, 34). Mechanical prophylaxis should be reserved for those patients who have an absolute bleeding risk or a relative bleeding risk where the risk of bleeding outweighs the risk of developing VTE. Step 3 (reassessment): to reassess the status of VTE parodically (e.g., each 3three days) or when the patient's condition changes (e.g., recurrence of stroke or occurrence of pneumonia, etc.) and feedback to modify DVT prevention strategies. With the R–P–R model, we look forward to improving ICH outcome by preventing DVT individually, effectively, and economically.

To the best of our knowledge, we are the first to derive and validate a risk score for predicting in-hospital DVT after ICH. The ICH-DVT score is unique in that it was derived from a large, multicenter, and prospective ICH cohort, which included consecutive patients of ICH, was outside of clinical trials, and was more reflective of real-world clinical practice. However, our study had some limitations that deserve comment. First, we only have information on new-onset DVT during hospitalization without documentation of the exact date of in-hospital DVT after ICH. Our data allow no conclusion as to whether patients with a longer length of stay *per se* are more likely to develop DVT or whether occurrence of DVT leads to a longer hospitalization. Second, the study included only hospitalized stroke patients and those patients died in emergency department, shortly after admission, or treated in outpatient clinics were not included. Third, the ICH-DVT needs to be further validated in additional populations and larger samples.

## Conclusion

The ICH-DVT is a valid grading scale for predicting in-hospital DVT after ICH. Further studies on the effect of the ICH-DVT on clinical outcomes after ICH are warranted.

## Research in context

### Evidence before this study

We did a systematic review of studies of prognostic model of spontaneous intracerebral hemorrhage published in OVID MEDLINE (from January 1, 1990, to December 31, 2020) using a comprehensive search strategy, limited to humans, combining terms for intracerebral hemorrhage (“intracerebral hemorrhage/,” “intracranial hemorrhages/,” “cerebral hemorrhage/,” “intracranial hemorrhage, hypertensive/,” and other text words) with key words suggesting deep vein thrombosis (DVT), venous thromboembolism (VTE), or pulmonary embolism (PE) prediction (“risk models,” “score,” “equation,” “predictive model”), with no language restriction.

Despite advances in medical knowledge, the treatment of ICH remains strictly supportive with not many evidence-based interventions currently available. Medical and surgical treatments, such as blood pressure control, hematoma evacuation, hemostatic therapy, and neuroprotection, have not shown a definite benefit in improving ICH functional outcome.

Venous thromboembolism (VTE) is a common and potentially life-threatening complication after stroke. VTE includes deep vein thrombosis (DVT) and pulmonary embolism (PE). The former is the most prevalent presentation, and the latter is the most severe form of VTE. Studies have indicated that patients with hemorrhagic stroke are at significantly higher risk of DVT than those with ischemic stroke. DVT prophylaxis might be a potential target to improve clinical outcomes after ICH.

Currently, no reliable scoring system is available to predict in-hospital DVT after ICH in routine clinical practice or clinical trials. An effective risk stratification model for in-hospital DVT after ICH would be helpful to identify high-risk patients and implement tailored preventive strategies. In addition, for clinical trials, it could be used in nonrandomized studies to control for case-mix variation and in controlled studies as a selection criterion.

### Added value of this study

To the best of our knowledge, we are the first to derive and validate a risk score for predicting in-hospital DVT after ICH. It was found that age (*P* < 0.001), hematoma volume (*P* = 0.01), subarachnoid extension (*P* < 0.001), pneumonia (*P* < 0.001), gastrointestinal bleeding (*P* = 0.003), and length of hospitalization (*P* < 0.001) were significantly associated with in-hospital DVT after ICH. A 31-point ICH-DVT score was developed from the set of independent predictors. The ICH-DVT showed good discrimination and calibration in the derivation (*n* = 1,309), internal validation (*n* = 655), and external validation (*n* = 315) cohorts. The predictive performance (AUROC) of the ICH-DVT in the derivation, internal validation, and external validation cohorts was 0.81 (95% CI = 0.79–0.83), 0.83 (95% CI = 0.80–0.86), and 0.88 (95% CI = 0.84–0.92). The Hosmer–Lemeshow test was not significant in derivation (*P* = 0.53), internal validation (*P* = 0.38), and external validation (*P* = 0.06) cohorts.

### Implications of all the available evidence

The ICH-DVT is a valid grading scale for predicting in-hospital DVT after ICH. The ICH-DVT score could be helpful to identify high-risk patients of developing in-hospital DVT after ICH, which would be useful for implementing tailored preventive strategies. In addition, for clinical trials, ICH-DVT could be used in nonrandomized studies to control for case-mix variation and in controlled studies as a selection criterion.

## Data availability statement

The raw data supporting the conclusions of this article will be made available by the authors, without undue reservation.

## Ethics statement

The studies involving human participants were reviewed and approved by Institutional Review Board (IRB) of the Beijing Tiantan Hospital (KY2014-023-02). The patients/participants provided their written informed consent to participate in this study.

## Author contributions

RJi and XZ: conception and design. XZ: administrative support. Beijing Registration of Intracerebral Hemorrhage investigators: provision of study materials or patients and collection and assembly of data. RZ, GL, and RJia: data analysis and interpretation. All authors: manuscript writing and final approval of manuscript.

## Funding

This study was sponsored by the Capital Health Research and Development of Special (2011-2004-03) and Beijing Municipal Science and Technology Commission (Z131107002213009). This study was partially supported by the Nova Program of Beijing Science and Technology Commission (2008B30), National Natural Science Foundation of China (81471208 and 81641162), Beijing High-Level Healthy Human Resource Project (014-3-033), and Shandong Province Key Innovation Project (2019JZZY020901).

## Conflict of interest

The authors declare that the research was conducted in the absence of any commercial or financial relationships that could be construed as a potential conflict of interest.

## Publisher's note

All claims expressed in this article are solely those of the authors and do not necessarily represent those of their affiliated organizations, or those of the publisher, the editors and the reviewers. Any product that may be evaluated in this article, or claim that may be made by its manufacturer, is not guaranteed or endorsed by the publisher.
