# Supplementary material for: A novel risk score to predict deep vein thrombosis after spontaneous intracerebral hemorrhage
Source: Front Neurol. 2022 Oct 28;13:930500. doi: 10.3389/fneur.2022.930500 (PMC9650187; doi:10.3389/fneur.2022.930500)
Supplement: Supplementary file 1 [file Data_Sheet_1.docx]

**Supplementary Table 1. Univariable predictor of in-hospital DVT after ICH in the derivation cohort (n=1309)**

|  | **Increment/categories** | **OR** | **95% CI** | **P value** |
| --- | --- | --- | --- | --- |
| Demographics |  |  |  |  |
| Age, y | 1 year increase | 1.03 | 1.02-1.05 | <0.001 |
| Gender (male) | female vs. male | 1.23 | 0.84-1.81 | 0.28 |
| Onset to hospital (hours) | 1 hour increase |  |  |  |
| Risk factors |  |  |  |  |
| Hypertension | yes vs. no | 0.89 | 0.60-1.31 | 0.55 |
| Diabetes mellitus | yes vs. no | 1.22 | 0.75-1.99 | 0.42 |
| Dyslipidemia | yes vs. no | 1.28 | 0.72-2.27 | 0.41 |
| Atrial fibrillation | yes vs. no | 2.37 | 0.81-6.89 | 0.11 |
| History of stroke/TIA | yes vs. no | 0.92 | 0.55-1.55 | 0.76 |
| Myocardial infarction | yes vs. no | 2.48 | 0.34-18.2 | 0.37 |
| Heart failure | yes vs. no |  |  |  |
| Current smoker | yes vs. no | 1.04 | 0.70-1.55 | 0.84 |
| Alcohol consumption | yes vs. no | 0.91 | 0.62-1.33 | 0.62 |
| Pre-admission anticoagulation | yes vs. no | 1.59 | 0.37-6.95 | 0.53 |
| Pre-admission antiplatelet | yes vs. no | 1.06 | 0.63-1.78 | 0.83 |
| Pre-stroke mRS score | 1 grade increase | 0.83 | 0.63-1.12 | 0.23 |
| Admission NIHSS score | 1 point increase | 1.02 | 1.00-1.03 | 0.07 |
| Admission GCS score | 1 point increase | 0.96 | 0.92-1.00 | 0.07 |
| Admission dysphagia | yes vs. no | 2.44 | 1.68-3.53 | <0.001 |
| Admission SBP (mm Hg) | 1 mm Hg increase | 1.00 | 0.99-1.00 | 0.33 |
| Admission DBP (mm Hg) | 1 mm Hg increase | 0.99 | 0.98-1.00 | 0.08 |
| Admission WBC, 10^9^/L | 1×10^9^/L increase | 1.00 | 0.99-1.01 | 0.87 |
| Admission glucose (mmol/L) | 1×mmol/L increase | 1.00 | 0.99-1.01 | 0.93 |
| Admission creatinine (umol/L) | 1×ummol/L increase | 0.99 | 0.98-1.00 | 0.05 |
| Hematoma location | Supratentorial vs. Infratentorial | 2.06 | 0.95-4.47 | 0.07 |
| Hematoma volume (cm^3^) | 1 ml increase | 1.01 | 1.00-1.02 | <0.001 |
| Intraventricular extension | yes vs. no | 1.82 | 1.26-2.63 | 0.001 |
| Subarachnoid extension | yes vs. no | 2.59 | 1.68-3.95 | <0.001 |
| Etiology diagnosis | Primary ICH vs. IVH | 1.25 | 0.17-9.41 | 0.83 |
|  | Secondary ICH vs. IVH | 1.41 | 0.17-11.6 | 0.75 |
| Ambulatory within 48h after admission | yes vs. no | 0.40 | 0.23-0.70 | 0.001 |
| ICP for DVT prophylaxis within 48h after admission | yes vs. no | 1.61 | 0.79-3.28 | 0.19 |
| Anticoagulation for DVT prophylaxis within 48h after admission | yes vs. no | 3.79 | 0.42-34.2 | 0.23 |
| Withdrawal of medical care | yes vs. no | 0.67 | 0.29-1.53 | 0.34 |
| Surgical treatment | yes vs. no | 2.46 | 1.66-3.64 | <0.001 |
| length of hospital stay | 1 day increase | 1.02 | 1.01-1.03 | <0.001 |

**Supplementary Table 2. Calibration of ICH-DVT with regard to in-hospital DVT after ICH**

| **Cohort** | **Goodness of fit test with regard to SAP after ICH** | | |
| --- | --- | --- | --- |
|  | P value | Cox and Snell R^2^ | Nagelkerke R^2^ |
| Derivation cohort (n=1309) | 0.53 | 0.07 | 0.20 |
| Internal validation cohort (n=655) | 0.38 | 0.08 | 0.22 |
| Overall cohort (n=1964) | 0.61 | 0.08 | 0.21 |
| External validation cohort (n=314) | 0.06 | 0.11 | 0.32 |

**Supplementary Table 3.** **Multivariable predictors of in-hospital DVT after ICH (without length of hospital stay) in the derivation cohort (n=1309)**

| **Variables** | **β-coefficients** | **SE** | **adjusted OR*** | **95% CI** | **P** |
| --- | --- | --- | --- | --- | --- |
| Model intercept | -4.795 |  |  |  |  |
| Age (1 year increase) | 0.025 | 0.007 | 1.025 | 1.010-1.040 | <0.001 |
| Hematoma volume (1 ml increase) | 0.006 | 0.002 | 1.006 | 1.001-1.011 | <0.01 |
| Subarachnoid extension (yes) | 0.949 | 0.237 | 2.583 | 1.623-4.113 | <0.001 |
| Occurrence of pneumonia (yes) | 1.274 | 0.234 | 3.574 | 2.261-5.650 | <0.001 |
| Occurrence of GIB (yes) | 0.887 | 0.248 | 2.429 | 1.493-3.952 | <0.001 |

*Multivariable logistic regression adjusted for demographics, time from onset to hospital, stroke risk factors, pre-admission antithrombotic medications, pre-stroke dependence, admission NIHSS and GCS score, blood pressure, blood glucose, hematoma volume, location, intraventricular and subarachnoid extension, etiology, ambulation within 48h after admission, DVT prophylaxis within 48 hours after admission, surgical treatment, withdrawal of medical care and in-hospital medical complications.

Abbreviation: DVT, Deep Vein Thrombosis.; ICH, Intracerebral Hemorrhage; SE, Standard Error; OR, Odds Ratio; CI, Confidence Interval; NIHSS, National Institutes of Health Stroke Scale; GCS, Glasgow Coma Scale; GIB, gastrointestinal bleeding.

The predictive performance of the risk model in the derivation (n=1,309) and internal validation cohort (n=655) was 0.744 (95%CI=0.659-0.829) and 0.749 (95%CI=0.567-0.931), respectively.

|  |  |
| --- | --- |
|  |  |
| **Supplementary Figure 1. Plot of observed versus predicted risk of DVT after ICH in the derivation and validation cohorts** | |

**Supplementary Figure 1 Legend**

Plot of observed versus predicted risk of in-hospital DVT after ICH in the derivation, internal and external validation cohort according to 10 deciles of predicted risk. Overall, there was a very high correlation between observed and predicted risk in the derivation cohort (A) (n=1309; r=0.97, P<0.001), internal validation cohort (B) (n=655; r=0.99, P<0.001), overall cohort (C) (n=1964; r=0.98, P<0.001) and external validation cohort (D) (n=314; r=0.97, P<0.001), which indicated excellent calibration.
